# Immunohistochemical determination of estrogen and progesterone receptors in breast cancer: relationship with clinicopathologic factors in 302 patients in Ivory Coast

**DOI:** 10.1186/s12885-017-3105-z

**Published:** 2017-02-07

**Authors:** Ahoua Benjamin Effi, Nguiessan Alphonse Aman, Baumaney Sylvanus Koui, Kouadio Donatien Koffi, Zie Cheick Traoré, Mohamed Kouyate

**Affiliations:** 1 Department of Anatomic Pathology, School of Medicine, Alassane Ouattara University, BP V 18 Bouake, Ivory Coast; 2 Department of Anatomic Pathology, Treichville Teaching Hospital, 01 BP V 03 Abidjan 01, Ivory Coast

**Keywords:** Breast cancer, Estrogen receptor, Progesterone receptor, Immunohistochemistry, Clinicopathologic factors

## Abstract

**Background:**

Breast cancer is a heterogeneous and a hormone-dependent disease. The detection of the estrogen receptor (ER) and progesterone receptor (PgR) is crucial for prognostic evaluation and treatment choice of breast cancer for clinical practice. The purpose of this study was to evaluate the expression of the hormonal receptors, their distribution, and their correlation with clinicopathologic prognostic parameters for the improvement of the patients’ treatment in Ivory Coast.

**Methods:**

The 20-month prospective study included 302 patients who were diagnosed with primary invasive breast carcinomas at the Central Laboratory in Abidjan. The paraffin-embedded blocks of these patients were examined by immunohistochemistry to assess the ER and PgR status. The one-way analysis of variance and Chi-Square Test were used to analyze the data.

**Results:**

The mean age of patients at diagnosis was 48 ± 11 years. The majority of the women were premenopausal in 180 cases (59.9%). The predominant histologic type was invasive ductal carcinoma not otherwise specified (IDC NOS) in 247 cases (82%). Tumor grade 2 was more frequent in 166 cases (55%). Among 302 patients, 169 (56%) and 154 (49%) expressed ER and PgR respectively. The ER+PgR+ group with 131 cases (43%) was predominant, followed by 116 cases (38%) of ER-PgR-. The expression of ER and PgR was correlated with the age of the patients (*p* = 0.026) and the tumor grade (*p* = 0.0004). However, there was not statistically significant correlation between ER/PgR and the menopausal status of patients (*p* = 0.149), nor between ER/PgR and the histologic type (*p* = 0.523).

**Conclusion:**

The ER+PgR+ and ER-PgR- are the most common subgroups in women with breast cancer in Ivory Coast. The hormonal receptor status is associated with the age and the histologic grade in breast cancer patients. The systematic use of hormonal treatment should be reevaluated. A further study should be done to investigate the reasons of high rate of ER-PgR- in breast cancer patients in Ivory Coast.

## Background

Breast cancer is the most frequent malignant tumor and the most common cause of cancer-related death among women in the developed countries [1, 2]. Breast cancer is increasing in the developing countries, including Ivory Coast, where it ranks at the first cancer in women after cervical cancer [3]. Breast cancer is a hormone-dependent disease, and thus, resulting from the mitogenic effects of estrogen and progesterone [4, 5]. The positivity of the ER is generally more than 70% in women with breast cancer than that of PgR, 50% [6, 7]. The ER/PgR status is essential for clinical and therapeutic care of the breast cancer patients [8, 9]. The ER has well-established prognostic and predictive values [9, 10], while the PgR has a controversial additional predictive value [11, 12]. The presence or not of ER and PgR helps determine a possible relapse of breast cancer [9]. The hormonal receptor status allows to distinguish four subgroups of breast cancers: ER+PgR+, ER+PgR-, ER-PgR+, and ER-PgR- [8, 13, 14]. This classification helps to decide hormonal treatment for ER/PgR positive patients and chemotherapy for the ER/PgR negative patients [9, 15]. Although the immunohistochemical evaluation of ER and PgR is a routine clinical practice in the diagnosis and treatment of breast cancer management worldwide, the clinical utility of ER and PgR testing in breast cancer is currently performed since June 2013 in Ivory Coast. Moreover, very few studies have been done on small sample size (22 patients) to assess the hormonal receptor status of breast cancer in Ivory Coast [16]. The current research is essential to update the immunohistochemical activity of ER/PgR in primary breast cancers. Herein, the aim of this study was to evaluate the expression of ER and PgR, their distribution, and their correlation with classic clinicopathologic prognostic parameters (age, menopausal status, histologic type, and grade) to enhance the breast cancer patients’ medical care. The present study will contribute to classify patients into different subgroups based on their hormonal receptor status in order to determine the better treatment strategies for women with breast cancer in Ivory Coast.

## Methods

### Patients

The prospective study was conducted between November 2013 and June 2015, including 302 patients diagnosed with primary invasive breast carcinomas at the Central Laboratory in Abidjan, Ivory Coast. The histologic diagnosis was performed upon paraffin-embedded breast tissue blocks sampled from 261 (86.4%) needle core biopsies and 41 (13.6%) mastectomies. On each sample, the histologic type and the Nottingham grade of the tumor were determined according to the criteria of Elston and Ellis [17]. The parameters of the study were classic clinicopathologic parameters (age, menopausal status, histological type, and tumor grade) and the status of ER and PgR. Paraffin-embedded blocks of breast tissue were subjected to the immunohistochemical assessment.

### ER/PgR immunohistochemical analysis

The immunohistochemical analysis was performed on 3 μm thickness of breast tissue sections. Tissue sections were deparaffinized and heated in the drying oven BINDER ® (BINDER Company, Tuttlingen, Germany) for at least 12 h at 60^0^ C to unmask the antigenic sites. The sections were stained using the Ventana BenchMark ® GX in automatic mode (Ventana Medical Systems Inc., Tucson, AZ, USA) for the assessment of ER and PgR status. The antibody clones were monoclonal, developed in rats, consisted of SP1 for the ER and 1E2 for the PgR, and manufactured by Ventana Medical Systems, Inc.

### Staining assessment of ER/PgR

The visual analysis through the optic microscope allowed to evaluate the staining intensity (weak, moderate, intense) and the percentage of tumor cells showing a nuclear immunostaining for ER and PgR (range: 0-100%). Breast tissue sections were considered positive for the ER and PgR if ≥ 1% of tumor cells displayed a positive nuclear staining in accordance with the recommendations of the American Society of Clinical Oncology/College of American Pathologists [18]. The immunostaining intensity and the percentages of stained cells for ER and PgR were reviewed independently by two pathologists. For the purpose of this study, the percentages of tumor cell nuclei positively stained for ER and PgR were considered.

### Statistical analysis

Data were collected in an Excel database from Windows 8 (Microsoft Corporation, Redmond, WA, USA). The difference between the subgroups based on the status of ER/PgR and the mean age were evaluated by the one-way analysis of variance. The Chi-Square Test was used to analyze associations between classic clinicopathologic parameters (menopausal status, histological type, and tumor grade) and combined ER/PgR status. A p-value < 0.05 was considered statistically significant. The data were reported as frequencies for menopausal status, histological type, tumor grade, and ER/PgR status and as means for patients’age.

## Results

### Classic clinicopathologic parameters

The mean age of patients at diagnosis was 48 ± 11 years (extremes: 24–84 years). The clinicopathologic characteristics in breast cancer patients are shown in the Table 1. Among 302 patients, 180 cases (59.9%) were premenopausal patients compared to 112 cases (37.1%) of postmenopausal patients. The frequent histological type was IDC NOS with 247 cases (82%). Of these cancers, 41 (13.6%) were grade 1; 166 (55%) were grade 2; and 63 (20.9%) were grade 3.

**Table 1:** Frequency of clinicopathologic characteristics in breast cancer patients

Characteristics	*N* (%)
Menopausal status	
Premenopausal (<50 years)	180 (59.6)
Postmenopausal (≥50 years)	112 (37.1)
Histologic type	
IDC NOS	247 (82)
Lobular	13 (4.3)
Other	28 (9.3)
Tumor grade	
1	41 (13.6)
2	166 (55)
3	63 (20.9)
Unknown	32 (10.6)

### Hormonal expression of ER and PgR

The Table 2 summarizes the combined ER/PgR status. ER and PgR positivity was 169 cases (56%) and 148 cases (50%) respectively. Almost half (131 cases; 43%) of the women in this study expressed both ER and PgR, followed by 116 cases (38%) in ER-PgR- patients.

**Table 2:** Distribution of ER/PgR in breast cancer patients

Receptor status	*N* (%)
Estrogen receptor status	
Positive	169 (56)
Negative	133 (44)
Progesterone receptor status	
Positive	148 (49)
Negative	154 (51)
Estrogen/Progesterone receptor status	
ER+PgR+	131 (43)
ER+PgR-	38 (13)
ER-PgR-	116 (38)
ER-PgR+	17 (6)

### Association of ER/PgR status with classic clinicopathologic parameters

A significant relationship was found between the ER/PgR status and the age of patients (*p* = 0.026); and between the ER/PgR status and the tumor grade of breast cancer (*p* = 0.0004), while the correlations of the ER/PgR status with the menopausal status (*p* = 0.149) and with the histologic type (*p* = 0,523) were not statistically significant (Table 3).

**Table 3:** Relationship between ER/PgR with age, menopausal status, histologic type, and grade in breast cancer patients

	ER+PgR+ (*N* = 131)	ER+PgR- (*N* = 38)	ER-PgR- (*N* = 116)	ER-PgR+ (*N* = 17)	*p* value
Age					*p* = 0.026
Mean (± SD)	46 (±11)	49,05 (±9.20)	49 (±10.92)	53 (±10.20)	
Extreme	24-83	33-72	27-84	34-74	
Menopausal status					*χ* ^2^ = 6.1 *p* = 0.175
Premenopause	88	23	61	8	
Postmenopause	40	14	49	9	
Histologic type					*χ* ^2^ = 5.2 *p* = 0.523
IDC NOS	104	29	100	14	
Lobular	8	1	3	1	
Other	12	6	8	2	
Tumor grade					*χ* ^2^ = 18.9 *p* = 0.0004
1	26	5	10	0	
2	73	22	59	12	
3	20	4	36	3	

## Discussion

For decades, samples of patients diagnosed with invasive breast carcinomas were sent to laboratories equipped with immunohistochemical techniques in the developed countries for ER and PgR examination. The Roche-Hoffman Laboratory in Ivory Coast, in collaboration with the Ivorian Health Ministry, has recently offered a Unit of Immunohistochemistry to the Central Laboratory to investigate the ER/PgR status of breast cancer patients for an efficient medical support. This study aimed at determining the hormonal receptor status to better characterize breast cancer subtypes and to assess the association of the hormonal receptor with age, menopausal status, histologic type, and tumor grade.

In the present study, several significant observations have been identified. The mean age of all patients at the diagnostic was 48 years, indicating that breast cancer appears early, before the menopause. This finding is similar to several studies conducted in Africa [16, 19–22] and in the Middle East [23]. However, the mean age of our patients is different from that of the developed countries [2, 6, 24], where breast cancer commonly occurs at the advanced age or at the postmenopausal period. The early occurrence of breast cancer in women in Ivory Coast could be due to the relative short life expectancy (54 years), the multiparity, and the early age at first childbirth. Parkin et al. found that the multiparity increased the risk of breast cancer before 45 years in a study in Zimbabwe [25]. Moreover, the multiparity [26, 27] and the early age at first childbirth [28] were the main risk factors for breast cancer in black American women. These observations may explain the high incidence of the breast cancer in premenopausal patients in our study.

In this study, IDC NOS associated with tumor grade 2 was predominant. These results are in agreement with data of other studies [7, 29], suggesting that clinical prognostic factors of breast cancer are worse in the African women, including Ivorian women. In contrast, the histologic type and the tumor grade have insufficient prognostic and predictive implications with limited clinical utility [30]. Therefore, it is valuable to detect ER and PgR status immunohistochemically in the current study to evaluate the survival of patients and to select their treatment.

The proportion of patients expressing ER is superior to those of PgR+. The same finding was reported by different authors in Europe, [29, 31, 32], in the USA [7, 8], and in Africa [21, 33]. In addition, ER+PgR+ and ER-PgR- were the most frequent subtypes in the current study. Our remarks corroborate with results of several studies [8, 13, 27, 33], suggesting that ER+PgR+ patients should be considered for hormonal therapy, and ER-PgR- patients should benefit from chemotherapy. Previously, a large number of breast cancer women underwent a systematic hormonal treatment in a blind manner in Ivory Coast. However, 38% of the study patients may not suitable for hormonal therapy, tamoxifen, since they do not express ER and PgR. As a result, they will not benefit from hormonal therapy, and the chemotherapy remains the only systematic treatment [9, 34, 35]. In this study, ER+PgR+ patients would more favorably respond to hormonal therapy than ER+PgR- and ER-PgR+ patients [9, 14, 36]. Additionally, ER+PgR+ patients, receiving hormonal therapy, have the advantage of avoiding a tumor relapse leading to a good long-term survival [9].

The high rate of ER-PgR- in our study is a remarkable finding and is approximately comparable with results reported by Seshie et al. in Ghana [37], Galukande et al. in Uganda [38], Adeniji et al. in Nigeria [39], and Palmer et al. in black American women [27]. Palmer et al. identified that the ER-PgR- subtype is greatly aggressive and resistant to hormonal therapy whose incidence is increased in the black American population. This high rate is related to the multiparity [27] and the early age at first childbirth [28]. Further studies should be done to determine the inherent reasons of the large frequency of ER-PgR- patients in Ivory Coast. Additionally, the increased proportion of ER-PgR- subtype could be explained by a deficiency of the preanalytical factors, particularly the fixation quality, investigated by Werner et al. [40] and Goldstein et al. [41]. Hence, a multidisciplinary collaboration between oncologists, radiologists, and pathologists is required to have sampled breast tissues fixed within the allotted time (6–18 h) to preserve hormonal receptor epitopes [40, 41].

In this current study, the ER-PgR+ subtype, accounting for 6%, is identical to that reported by Osborne et al. [8] and Hefti et al. [13]; however, differ from that of Nadji et al. [7] and Inwald et al. [32], who listed 0% and 0.8% respectively. Hefti et al. [13] have recently found that ER-PgR+ group does not represent a subtype of biologically distinct or clinically important cancer, and therefore, should be regarded as a false negative. This artifact subtype results from an inappropriate fixation leading to the loss of epitopes of paraffin-embedded breast tissue blocks [7, 40, 41].

Despite the unquestionable contribution of ER and PgR testing for a better therapeutic implication, it appears necessary to examine the correlation between ER/PgR status with standard clinicopathologic parameters of primary invasive carcinomas in 302 patients. There was a significant association between the age of the patients and the ER/PgR subgroups. The ER/PgR status has no significant influence on the menopausal status. Our results are consistent with findings of Elwood and Godolphin. Both authors revealed in an analysis of multiple regression study of age and menopausal status in 735 patients that the mean age was significantly associated with the ER/PgR, while there was no significant link between the ER/PgR and the menopausal status [6]. In the past, tamoxifen was given based on the menopausal status of patients in Ivory Coast because postmenopausal patients appeared to be ER+PgR+ and would better respond to tamoxifen than premenopausal patients. As a result, our data pinpoint that hormonal therapy should be given regardless of the menopausal status.

A correlation was found between the ER/PgR and the tumor grade, which corroborates with the literature data [6, 7]. In contrast, there was not significant association between ER/PgR status and the histologic type. This result differs from finding of numerous studies [6, 7, 34]. Nadji et al. observed that ER status predicted some histologic types in breast cancer, and thereby, a lack of such correlation in our study should suspect a technical problem [7]. In our study, this technical problem may be resulted from the morphologic diagnostic errors or the handling issue of preanalytical factors of breast samples, such as the duration and the type of fixation.

## Conclusions

The ER+PgR+ and ER-PgR- are the most common subtypes and occur in premenopausal women. The hormonal receptor status is associated with the age and the tumor grade in breast cancer patients. Taken together, the results of this study help to eliminate a systematic use of hormonal therapy based on the menopausal status of the breast cancer patients. The increased proportion of ER-PgR- patients needs to be carefully considered in a future study. The ER/PgR status is no longer sufficient to treat breast cancer patients in Ivory Coast since the human epidermal growth factor receptor 2 (Her2) overexpression analysis and the Ki67 index are required to define the molecular classification of breast cancers for better treatment strategies.

## Abbreviations

ER: Estrogen receptor
Her2: Human epidermal growth factor receptor 2
IDS NOS: Invasive ductal carcinoma not otherwise specified
PgR: Progesterone receptor
